# Compound urban crises

**DOI:** 10.1007/s13280-021-01697-6

**Published:** 2022-02-14

**Authors:** Linda Westman, James Patterson, Rachel Macrorie, Christopher J. Orr, Catherine M. Ashcraft, Vanesa Castán Broto, Dana Dolan, Mukesh Gupta, Jeroen van der Heijden, Thomas Hickmann, Robert Hobbins, Marielle Papin, Enora Robin, Christina Rosan, Jonas Torrens, Robert Webb

**Affiliations:** 1 Urban Institute, University of Sheffield, 419 Portobello, Sheffield, S14DP UK; 2 Copernicus Institute of Sustainable Development, Faculty of Geosciences, Vening Meineszgebouw A, Princetonlaan 8A, 3585CB Utrecht, The Netherlands; 3 Transforming Cities Hub, Human Geography and Spatial Planning Department, University of Utrecht, Princetonlaan 8a, 3584 CB Utrecht, The Netherlands; 4 University of Waterloo, 200 University Avenue, Waterloo, ON N2L 3G1 Canada; 5 Department of Natural Resources & the Environment, University of New Hampshire, 134 James Hall, 56 College Road, Durham, NH 03824 USA; 6 Schar School of Policy and Government at, George Mason University, 3351 Fairfax Drive, Arlington, VA 22201 USA; 7 Department of Environmental Sciences, Asian University for Women, 20/A M. M. Ali Road, Chattogram, 4000 Bangladesh; 8 Wellington School of Business and Government of Victoria University of Wellington, Rutherford House, Pipitea Campus, Wellington, 6011 New Zealand; 9 Urban Studies Institute, Georgia State University, 55 Park Place NE, Atlanta, GA 30303 USA; 10 Department of Geography and Urban Studies at Temple University, 320 Gladfelter Hall, 1115 Polett Walk, Philadelphia, PA USA; 11 Institute for Climate, Energy and Disaster Solutions and Fenner School of Environment and Society, Australian National University, Canberra, ACT 2600 Australia; 12 Dep of Industrial Engineering and Innovation Sciences, Eindhoven University of Technology, PO Box 513, 5600 MB Eindhoven, The Netherlands; 13 Faculty of Social Sciences, Department of Political Science, Lund University, Allhelgona kyrkogata 14, 221 00 Lund, Sweden; 14 McGill University, 805 Rue Sherbrooke Ouest, Montréal, QC H3A 0B9 Canada

**Keywords:** Cities, Complex adaptive systems, Compound urban crises, Critical urban studies, Governance

## Abstract

The crises that cities face—such as climate change, pandemics, economic downturn, and racism—are tightly interlinked and cannot be addressed in isolation. This paper addresses compound urban crises as a unique type of problem, in which discrete solutions that tackle each crisis independently are insufficient. Few scholarly debates address compound urban crises and there is, to date, a lack of interdisciplinary insights to inform urban governance responses. Combining ideas from complex adaptive systems and critical urban studies, we develop a set of boundary concepts (unsettlement, unevenness, and unbounding) to understand the complexities of compound urban crises from an interdisciplinary perspective. We employ these concepts to set a research agenda on compound urban crises, highlighting multiple interconnections between urban politics and global dynamics. We conclude by suggesting how these entry points provide a theoretical anchor to develop practical insights to inform and reform urban governance.

## Introduction

From economic downturn and austerity to inequalities and racism, pandemics, rapid technological shifts, ecological crises, and political polarisation, challenges to sustainability are not only numerous but increasingly interactive. Addressing any of these crises in isolation is impossible. For example, the climate emergency and social equity crises are tightly interlinked (Long and Rice 2020). In addition, turbulence is becoming an ever more pervasive global phenomenon (Dauvergne and Shipton forthcoming), creating a renewed scholarly interest in crises, disasters, and emergencies in the context of sustainability politics (Patterson et al. 2021).

In this paper, we focus on cities as sites where multiple crises manifest. Urban areas have long been viewed as recipients of different forms of shocks, ranging from violence (Muggah and Savage 2012) to pandemics (Keil and Ali 2007). Recent calls to address the climate crisis reverberate through urban politics and give it a renewed sense of urgency (Ruiz-Campillo et al. 2021). Protests against social, racial, gender-based, and economic injustice have erupted in cities across the globe, stressing enduring problems that have reached unbearable proportions (e.g. Sehnbruch and Donoso Knaudt 2020). Cities also face a global biodiversity crisis, forced migration, and economic shocks exacerbated by eroding labour protections, low-paid work, and poverty (Haase et al. 2018). City authorities are expected to respond to these issues, often despite limited formal powers and ability to raise funds, fragmented governance systems, and (in some parts of the world) long-term erosion of governance capacities due to funding cuts and privatisation.

Two significant challenges are understanding these interconnected issues and delivering appropriate and equitable responses. There are, however, gaps in the knowledge on the phenomenon of co-occurring urban crises. First, an established research tradition addresses the interplay between drivers and outcomes of crises, especially the scholarships on transboundary crises (Boin 2019) and compound risk (Zscheischler et al. 2018). However, these theories are not grounded explicitly in urban perspectives. Crises have shaped cities through history, reflecting entrenched social and economic inequality patterns and injustices. Contemporary disruptions are conditioned within particular urban contexts that shape their form, consequences, and possible responses. Understanding these dynamics requires analytical lenses attuned to political and spatial dimensions, often not reflected in traditional crisis or risk theory. Indeed, scholars of crisis call for more nuanced frameworks (Mitroff et al. 2004), particularly concerning social and historical contexts that shape disruption (Quarantelli et al. 2018).

Second, urban studies scholarship has long attended to how systemic vulnerabilities and inequalities shape the reproduction of everyday life in cities (e.g. Lees 2012; Pulido 2017). Yet, this body of research is seldom in dialogue with complex systems analyses. Today, the underlying causes of disruption in cities originate far beyond any given urban territory. They may relate to multiple levels of governance, ecosystem disruptions in other parts of the world, broad social struggles (e.g. over rights and recognition), and global economic forces (e.g. international finance). There is a need for analytical perspectives that consider both historical constituents of urban vulnerabilities and novel mechanisms of reproduction of risk based on global interconnectivity. Thus, the objective of this paper is to formulate a conceptual approach that facilitates the exchange of insights into compound urban crises across disciplinary boundaries.

The paper combines two strands of scholarship: complex adaptive systems (CAS) and critical urban studies (CUS). The CAS literature offers insight into the systemic nature of compound crises, while debates in CUS highlight asymmetric impacts on social groups across diverse settings. Despite their mutual interests, interchanges between these two bodies of work have been rare. Our analysis identifies entry points to examine convergences and complementarities between CAS and CUS. We think of these entry points as boundary concepts, that is, as anchors that facilitate interdisciplinary dialogue, shared vocabularies, and joint knowledge production. Boundary concepts are “words that operate as concepts in different disciplines or perspectives, refer to the same object, phenomenon, process or quality of these, but carry (sometimes very) different meanings in those different disciplines or perspectives” (Mollinga 2008, p. 25). Boundary concepts create bridges between literatures that address similar concerns, yet are not in dialogue. We deploy boundary concepts strategically to create a cognitive space where the contrasts between different systems of signification can be examined. The three boundary concepts for the study of compound urban crises that facilitate the interchange between CAS and CUS are *unsettlement* (enduring disruption of governance systems and everyday lives), *unevenness* (differentiated impacts across diverse societies), and *unbounding* (indeterminate problem boundaries and interactions).

The paper proceeds as follows. In ‘Conceptualising compound urban crises’, we map the theoretical foundation for compound urban crises and elaborate on the three boundary concepts. In the following section, ‘A research agenda for the study of compound urban crises’, we outline a research agenda indicated by these boundary concepts, highlighting the interconnections between global dynamics and the politics of urban precarity, the reproduction of structural injustice in cities, and the challenge of achieving knowledge pluralism. Finally, we reflect on the prospects of boundary concepts to stimulate exchange across scholarly divides.

## Conceptualising compound urban crises

### From single to compound crises

A crisis involves uncertainty, urgency, and threats to fundamental social structures or values (Farazmand 2001; Boin et al. 2016). In this paper, we understand urban crises as “a continuum where chronic vulnerabilities or structural states of crisis can themselves lead to episodic moments of acute shocks” (Robin et al. 2019), which destabilise operations and require urgent responses.

**Fig. 1:**
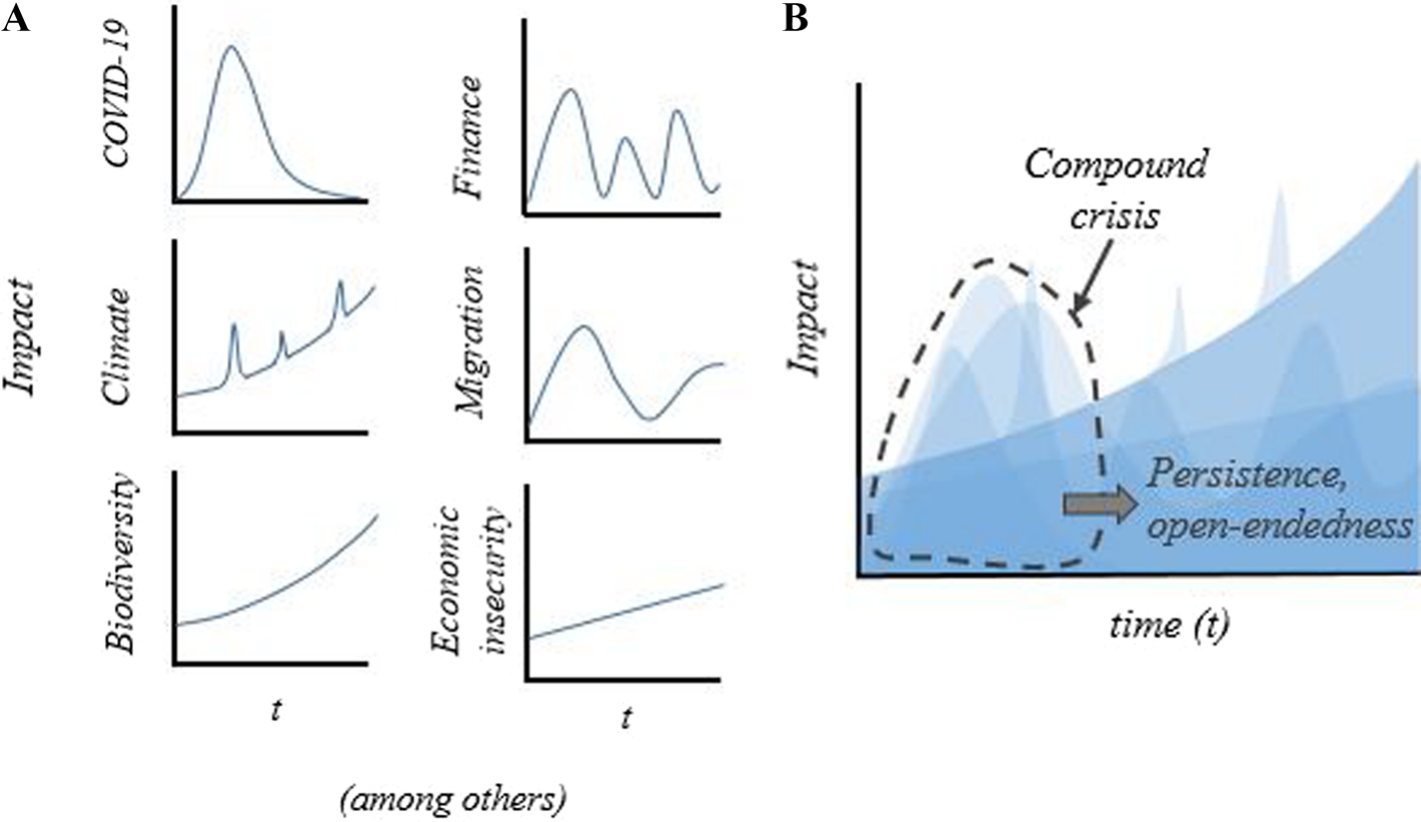
Multiple crises facing cities, viewed as either **A** singular crises, or **B** compound crises based on differences in temporal dynamics

In the context of disruption in cities, treating crises as single processes or events is becoming increasingly untenable. Many contemporary urban crises cannot be untied from one another, and assigning causes, impacts, and responses is not a clear-cut task (Katz 2010). There is disagreement about relevant timeframes, spatial scales, and affected social groups. Figure 1 illustrates two different ways of viewing crises from a temporal perspective, as either singular or compound crises. For example, climate change is a phenomenon that develops over the longue durée (though manifesting through immediate disasters), which over time has accumulated to threaten global earth system functions. By contrast, the COVID-19 pandemic emerged as a sudden acute moment of shock (Fig. 1A). Crises of finance and migration commonly face cities and may be sporadic or chronic. A perspective of compound crises emphasises interconnections between these issues, overlapping and varying over time, creating bundles of interlinked challenges. Compound crises lack self-evident stopping rules for delineating the scope of attention; the interaction between issues cannot be ignored (Fig. 1B).

Crises are socially constructed phenomena ('t Hart 1993; Quarantelli et al. 2018), caused by multiple exogenous and endogenous factors (Pearson and Mitroff 1993). They seldom if ever occur singly, but instead in tandem or sequentially (Roux‐Dufort 2009). There is often a complex interplay between fast- and slow-moving variables, such as in ‘creeping crises’ (Boin et al. 2020). In particular, the concept of transboundary crises captures complex interactions of both causes and impacts ('t Hart et al. 2001; Ansell et al. 2010; Quarantelli et al. 2018; Boin 2019). Transboundary crises cross national boundaries and policy domains, incubate before rapidly escalating, may lack a clear beginning and end, defy efforts to identify causes, consequences, and trajectories, involve multiple actors with competing goals, and lack readily apparent solutions (Ansell et al. 2010; Quarantelli et al. 2018; Boin 2019).

Likewise, the concepts of compound and cascading risk capture the interaction of drivers (in multiple sectors), across multiple timeframes (fast- and slow-moving) and geographies (proximate and remote), which creates complex, non-linear, and unpredictable dynamics (Wahl et al. 2015; Zscheischler et al. 2018). The concept of compound risk, applied in climate change research, is interpreted by the Intergovernmental Panel on Climate Change as the spatial convergence of impacts in different sectors, which leads to extreme or high-risk consequences (Oppenheimer et al. 2014, p. 1057). Table 1 contrasts notions of singular crises, transboundary crises, and compound risks based on their temporal, spatial, and sectoral dimensions.

**Table 1:** Causes and impacts in studies with a framing of singular crisis (with examples from Covid-19), transboundary crises, and compound risks (drawing on the climate change literature)

Framing	Temporal	Spatial	Sectoral
Singular crisis	Crisis onset as a sudden moment when *causes* are recognised and *impacts* become salient (Buchheim et al. 2020)	A single root *cause* is assumed (Li et al. 2020) that produces *impacts* in specific locales (Kim and Bostwick 2020)	*Causes* and *impacts* are framed within discrete sectors, such as health (Sohrabi et al. 2020) or housing (AI-Dafar 2020)
Transboundary crisis	The *causes* and *impacts* may be indeterminate (no clear beginning and end) (Ansell et al. 2010)	There is no ‘ground zero’ (Boin 2019) or ‘point of origin’ (Quarantelli et al. 2018) in terms of *cause*, while *impacts* transcend geographical, political, and legal boundaries (Boin 2019)	*Causes* and *impacts* manifest in different sectors/policy domains, involving multiple actors and conflicting responsibilities (Boin 2019)
Compound risks	Compound risks involve multiple *causes* (Wahl et al. 2015; Zscheischler et al. 2018). *Impacts* unfold on multiple temporal scales (Zscheischler et al. 2018)	Compound risks arise from the interaction of multiple *causes* at different spatial scales or governance levels and generate *impacts* across spatial scales (Zscheischler et al. 2018)	Compound risks arise from *causes* that cut across sectoral boundaries and *impacts* multiple sectors (Oppenheimer et al. 2014)

In conceptualising compound urban crises, we recognise that causes and impacts cut across temporal, spatial, and sectoral bounds. In contrast with the literature on transboundary crises, we locate the challenge of compound urban crises beyond the bounds and logics of the state. While the concept of transboundary crises was introduced to capture problems that escape institutional borders, the analysis departs from the limits (and opportunities) of central government institutions (Ansell et al. 2010; Boin 2019) or the global character of crises (Quarantelli et al. 2018). We argue that disruption in urban areas represents a more confounding challenge that always involves multiple levels of authority and spaces beyond the reach of formal institutions (e.g. informal settlements). We contend that, unlike compound risk, a focus on compound crises brings us closer to how people experience disruption as events in their everyday lives. The ‘everydayness’ of crises in urban areas is a pervasive phenomenon (Kaika 2012; Bhattacharyya 2015). The focus on events also directly motivates thinking on implications for urban governance, as crisis events call for urgent responses.

### The interdisciplinary foundation of compound urban crises

Drawing on the insights of interconnectivity and non-linearity proposed by the compound risk literature, we argue that CAS theory can help understand compound urban crises. CAS theory was established within ecosystems studies and has gained prominence in social-ecological systems (SES) research and resilience studies (for an extensive review, see among others Lansing 2003; Levin et al. 2013; Preiser et al. 2018)). CAS theory emphasises a view of open systems comprising multiple interconnected elements across scales (Turner and Baker 2019; Orsini et al. 2020). Such systems change through adaptive cycles, which involve phases of continuity when rules are maintained, moments of abrupt crisis, and reorganisation (Walker et al. 2020). In essence, CAS adds the notion of adaptive capacities to traditional systems theory. CAS theory recognises that systems evolve in response to changes in their context, *and* that system constituents ‘remember’ and learn from previous configurations. Therefore, past changes influence the trajectory of future system change (Preiser et al. 2018). The idea that systems are potentially ‘manageable’ or ‘controllable’ in part explains the interest in CAS from SES scholars (Walker et al. 2004; Levin et al. 2013).

A strand of the CAS scholarship that engages explicitly with disruption is the literature on resilience. The concept of resilience has diverse intellectual origins and is conceptualised in a plurality of ways in theory and practice (Muñoz-Erickson et al. 2021). In the SES literature, resilience is defined as the “capacity of a system to absorb disturbance and reorganize while undergoing change so as to still retain essentially the same function” (Folke et al. 2010). The SES scholarship associates certain system attributes with high levels of resilience, such as diversity, opportunities for collaboration and social learning, self-organisation, reflexivity, and interaction across scales (Olsson et al. 2004; Lebel et al. 2006). This literature also explains that crises provide opportunities for social and institutional renewal and innovation (Walker et al. 2020). This notion is captured by the concept of transformation, which explains reconfiguration of essential system functions in response to ecological or social conditions that have become untenable (Walker et al. 2004).

Resilience theory has moved beyond academic debates to function as a framework and discourse that shapes policy and action in cities. As a concept that engages with multiple forms of risk, resilience can inspire interventions that strengthen social protection, disaster risk management, and ecosystems in cities (Ziervogel et al. 2017; Borie et al. 2019; Khirfan and El-Shayeb 2020). In collaborative and community-driven resilience projects, such interventions can be aligned with local priorities and open new spaces of experimentation, knowledge exchange, and social learning (Orleans Reed et al. 2013; Bahadur and Tanner 2014; Fastenrath and Coenen 2021). For example, the 100 Resilient Cities initiative, spearheaded by the Rockefeller Foundation, has created a variety of technical tools, including a preliminary resilience assessment and agenda-setting workshops, aimed to facilitate the development and adoption of resilience strategies in its member cities (Nielsen and Papin 2021). However, the mobilisation of resilience discourse in urban policy can also serve as a superficial branding exercise (Dolan et al. 2010; Van der Heijden 2017), which fails to address structural drivers of vulnerability. In particular, urban resilience programs often struggle to shift political–economic structures that generate marginality and exclusion in cities (Friend and Moench 2013; Chu and Michael 2019; Weinstein et al. 2019). For example, a recent review of the 100 Resilient Cities program found that plans generally neglect procedural and recognitional dimensions of equity, even though these aspects are essential to address underlying structural drivers of vulnerability, such as systemic racism (Meerow et al. 2019).

Similarly, there are concerns that some forms of CAS analysis overlook the situated nature of human–environment relations (Cote and Nightingale 2012; Olsson et al. 2015) and may not sufficiently address political dimensions, such as emerging concerns about resilience as a form of resistance (Rivero-Villar 2021). For example, the uncritical application of discourses of transformation in urban environments may yield limited change, or even reinforce dominant political–economic structures (Westman and Castán Broto 2021, Forthcoming). CAS theory provides only limited insight into urban politics, including questions of agency and normative perspectives on urban development (Berkes and Ross 2013; Nel et al. 2018). Hence, a dialogue with critical urban studies (CUS) can inform and expand the research interests in CAS.

CUS raises issues that complement CAS theory. CUS does not offer a unified theoretical framework, but brings together different strands of work offering a critical perspective on how power relations, structural inequalities, cultural norms, forms of knowledge, values, and worldviews shape cities’ political and social fabric, urban economies, urban infrastructures, and the built environment (Graham and Marvin 2002; Simone 2018; Castán Broto et al. 2020). CUS complements CAS by focussing on relations, processes of urban change, and their spatial manifestations. Within CUS, the city represents a “nexus of trans-local and post-human flows of people, investments, policies, and matter” (Lancione 2019, p. 183). This perspective invites us to think of the city as a processual and heterogeneous configuration of material elements (e.g. roads, dust, buildings, trains, rubbish), formal and informal institutions (e.g. laws, regulations, taxes), and beings (e.g. plants, animals, humans), all of which interact at multiple scales, producing variegated effects (McFarlane 2011). Like CAS, CUS recognises the open-ended nature of these interactions and the difficulty in predicting their outcomes. CUS research on population displacements and housing struggles (Lees 2012; Ghertner 2015; Anguelovski et al. 2019; Lancione 2020), environmental racism (Pulido 2017), and urban climate action (Bulkeley et al. 2014; Shi et al. 2016), to name only a few, provides insights into how enduring and episodic moments of crisis, as well as responses to those, unevenly impact urban dwellers.

### Boundary concepts for the study of compound urban crises

**Fig. 2:**
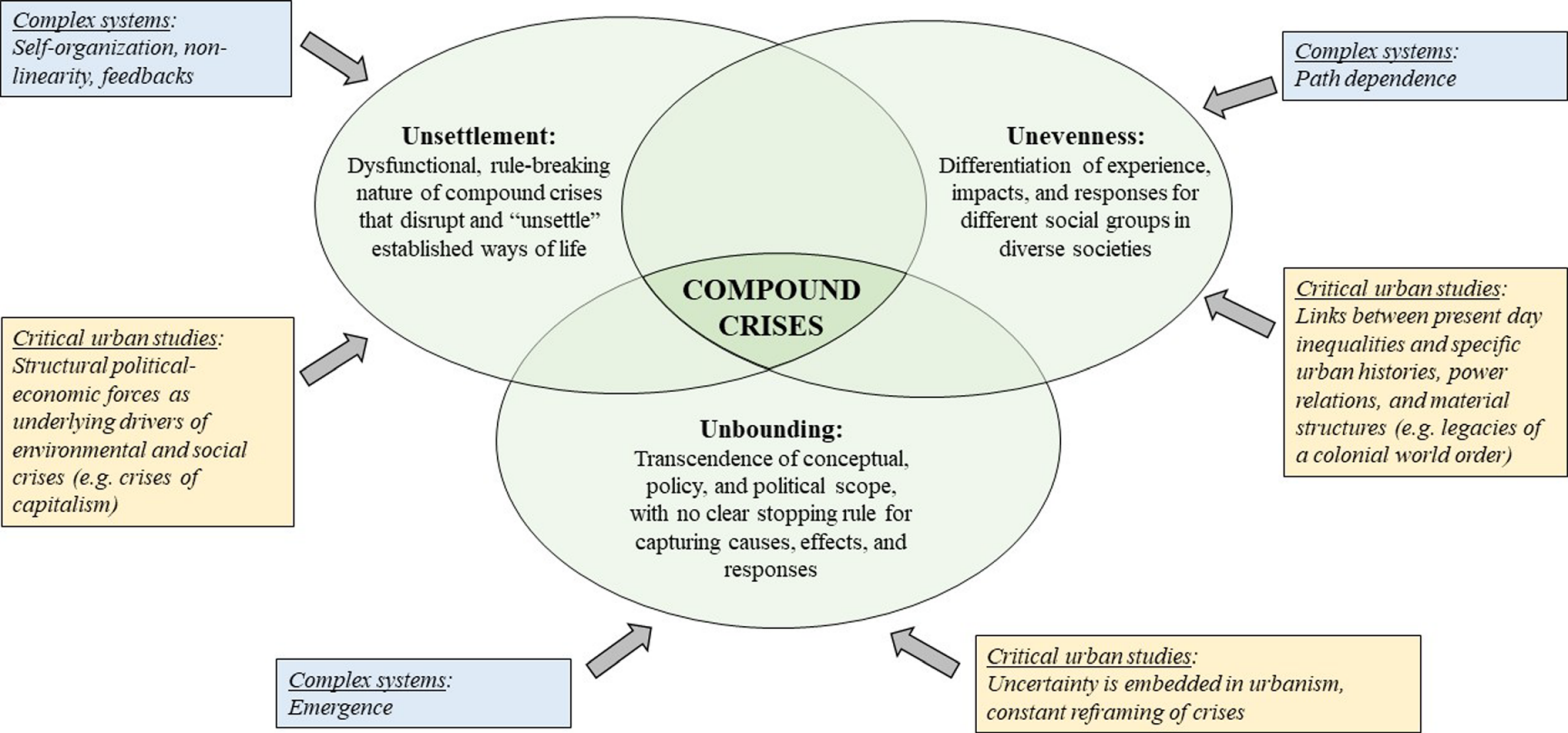
Conceptualising urban governance challenges associated with compound urban crises according to three key boundary concepts (unsettlement, unevenness, unbounding), drawing on insights from CAS and CUT

We introduce three boundary concepts that can build dialogue across CAS and CUS: unsettlement, unevenness, and unbounding. Table 2 summarises our conceptualisation of these boundary concepts, also captured by the exploratory heuristic in Fig. 2. Below, we explain how these boundary concepts highlight complementary insights from CAS and CUS into compound urban crises.

**Table 2:** Boundary concept definitions and summary of complementary insights brought by CAS and CUS

	Definition	CAS	CUS
Unsettlement	A state in which compound crises come to “unsettle” governance processes and everyday ways of life (Orr 2020). Such destabilisation disrupts established practices and systems of material and social support	CAS theory explains how unsettlement arises from the interconnected nature of complex systems, with destabilising drivers and feedbacks that are difficult to anticipate and control	CUS recognises that structural political–economic forces create a permanent state of risk and instability in urban life for millions of people worldwide (Schilling et al. 2019)
Unevenness	The differentiation of experiences, impacts, and responses of compound urban crises across groups in diverse societies. Both compound crises themselves, as well as governance responses, contribute to unevenness	From a CAS perspective, unevenness relates to the principle of path dependency, which explains how differentiation within a system arises and persists over time	CUS highlights multiple historical drivers that produce and reproduce social categories of difference
Unbounding	The indeterminate conceptual and political scope of compound urban crises, which do not have clear stopping rules for delineating causes and effects and involve unanticipated interactions	The CAS literature highlights emergence as a key property of complex adaptive systems, which captures how the interaction between different forms of shocks creates entirely new and unpredictable phenomena	CUS emphasises that uncertainty is an inherent condition of urban life, but also that the framing of crises is a matter of social construction and power differentials

#### Unsettlement

The CAS literature highlights how unsettlement is associated with non-linear feedback dynamics inherent to complex systems that are self-organising (Meadows 2008). Non-linear feedback refers to conditions in which the size of inputs is disproportional to expected outputs (Turner and Baker 2019). Given the unpredictability and unintended consequences that non-linear feedback creates, interventions into any single domain may be inadequate to resolve the problems of a destabilised system. Unsettlement manifests in the entangled interactions between human and natural systems, illustrated, for example, by the impacts of climate change. Climate impacts disrupt everyday life in cities through extreme events (e.g. floods and landslides) and slow-moving stressors (e.g. water scarcity and population displacement) (Revi et al. 2014). These impacts ripple through supply chains and infrastructure networks in interconnected global systems, linking risks in widely dispersed locations with unpredictable and unintended impacts in different parts of the world (Serre and Heinzlef 2018).

CUS highlights the role of structural political–economic forces (e.g. capitalism and neoliberal models of development) as underlying drivers of persistent environmental and social crises in cities, which produce risk as a permanent condition of everyday urban life (Molotch and Logan 1984). Any barrier to the continued circulation of capital within and between cities generates instability which drives recurring shocks (Harvey 2011). The global capitalist system also produces enduring dysfunction. Many millions of people in cities worldwide live under constant threat and lack of security linked to the slow-moving crisis of capitalism and the dominance of informal, temporary, and insecure forms of work in the global economy (Amin 2010). Approximately 60% of the world’s workforce is employed precariously, mostly women (ILO 2018). This everyday insecurity is acutely visible in patterns of poverty, exclusion, dispossession, housing insecurity, and violence in cities (Gay et al. 2013; Vilenica et al. 2020). Set against the urban modernist ideal of a politically unified, socially equitable, and infrastructurally cohesive city (Zeiderman et al. 2017), the concept of unsettlement shows that precarity is not an exception, but an essential dimension of the contemporary urban condition.

Box 1 Example of unsettlement—COVID-19 and austerity An example of unsettlement is the connection between crises of austerity and the COVID-19 pandemic. The UK austerity programme, introduced in response to the global financial crisis of 2007–8, led to a series of deep public spending cuts coupled with tax increases, resulting in a stalling in the rise of life expectancy for the first time in a century (Marmot 2020). Alongside, the growth of the so-called ‘gig economy’ saw a rise in zero-hour and fixed-term contracts (European Parliament 2016), increasing the number of households living in precarious conditions. When COVID-19 restrictions were introduced in March 2020, they interrupted established support systems and coping mechanisms and had unintended consequences that exacerbated insecurities. The crisis had disproportionate impacts on groups relying on low-paid and insecure work, such as caring, leisure, and other service occupations. With little security, this precariat faces threats of destitution, ill health, or possibly death (Butler 2020).

**Unevenness**

According to CAS theory, system evolution is sensitive to initial conditions. The concept of path dependency explains how local rules develop within parts of a system, which shape dynamics as the system evolves (Levin 1998). While originally the concept described ecosystem dynamics, path dependencies also develop within institutional arrangements. The establishment of entrenched ways of thinking and doing, such as accepted management practices, can limit the ability to deal with crises. For example, path dependencies in public resource management institutions can hinder climate adaptation, contributing to the maintenance of social vulnerability (Barnett et al. 2015). In cities, path dependencies in institutions and spatial form can contribute to the cementation of inequalities over time, for example, through continued marginalisation of impoverished neighbourhoods (Wu et al. 2010). In the context of compound urban crises, the concept of path dependency explains patterns of vulnerability, as well as why reliance on established institutions may lead to unintended reproduction of inequalities.

CUS reveals links between present-day inequalities and specific urban histories, power relations, and material structures. For example, histories of colonisation, stigmatisation, and racial segregation have translated into patterns of economic and spatial inequalities in cities, including uneven access to services and decaying infrastructures (Njoh 2008; Picker 2017). Feminist and postcolonial perspectives highlight how urban space is structured according to histories and relations that reproduce white, heteronormative, and Eurocentric ideals (Peake 1993; Roy 2016), which reproduce inequalities. Urban inequalities are directly related to vulnerability to risks and differentiated impacts during crises. For example, climate change often impacts the urban poor more severely, especially those lacking access to housing and other basic infrastructures (Hardoy and Pandiella 2009). Compound urban crises elevate these threats as vulnerable groups are exposed to multiple threats at once.

Box 2 Example of unevenness—COVID-19 and racism An example of unevenness is the connection between the crises of COVID-19 pandemic and institutionalised racism. In the U.S., the Black Lives Matter protests were a response to police brutality and racial injustice. Yet, they also followed recognition among people of all races of the compounded racialised vulnerabilities associated with living in low-income urban areas and lacking access to protective amenities (Rosan and Heckert 2020). During COVID-19, these inequalities translated into higher vulnerability to the virus among African Americans (Abedi et al. 2020). In urban regions, measures to contain the spread of the virus exacerbated stigmatised and racialised groups’ vulnerability to state violence. In France, for example, citizens of banlieues (suburban low-income housing states) protested against police brutality, arrests, and fines to enforce lockdown measures.

**Unbounding**

The CAS literature highlights emergence as a key property of complex adaptive systems (Holland 1998). Emergence explains how at the scale of a system, the interplay of agents shapes a hidden but recognisable regularity in the behaviour of the whole system. System-level properties, characteristics, and patterns emerge from interactions between individual elements, which generate qualitatively different characteristics (Railsback 2001). This means that the behaviour of a system as a whole cannot be observed in, or reduced to, its parts (Mingers 2014). When considering compound urban crises, emergence highlights how the interaction between different forms of disruption creates entirely new and unpredictable phenomena. Interaction between different forms of shock (e.g. in terms of patterns of impact, persistence over time, or cycles of intensity) is set against the existing complexity of the city and diverse dynamics of disruption (including interactions across temporal, spatial, and sectoral scales). Compound urban crises are interconnected between human and natural systems, across sectors and traditional governance silos, confounding the prediction of events and outcome of responses.

CUS highlights that uncertainty is an inherent quality of contemporary urbanism, meaning that it is not necessarily a problem to solve or a disorder to correct (Zeiderman et al. 2017). The scholarship also recognises the constantly shifting framing and social construction of crises. For example, while climate change once was framed as an environmental issue that could be tackled through single-sector interventions, it has become understood as a manifestation of modernity and a fundamental part of social life itself (Bulkeley 2021). As a result, addressing climate change requires interventions within a growing number of sectors and reflection on everyday social practices. Similarly, the concept of resilience has expanded from a narrow focus on recovery from climate shocks to encompass the multiple political–economic structures that maintain inequalities and risk (Ziervogel et al. 2017). Given the diverse and unanticipated ways compound crises manifest, there is a political imperative to rethink the categories that structure urban governance.

Box 3 Example of unbounding—the shifting problem framing of COVID-19 An example of unbounding is the constantly shifting framing of the COVID-19 pandemic—initially as a health crisis and eventually as a social, economic, and environmental problem. There was initially a strong focus on the health consequences of the virus, pathways of contagion, and infection rates. However, over time it became clear that the pandemic connects to other policy areas. The climate crisis interacts with the pandemic through the confluence of climate hazards and virus outbreak (Phillips et al. 2020) and the reduction of GHG emissions through restrictions on economic activity (Le Quéré et al. 2020). Links are made with seemingly disparate issues, such as housing (AI-Dafar 2020), illustrating the inherently cross-cutting and evolving nature of COVID-19 as part of compound crises.

## A research agenda for the study of compound urban crises

These three boundary concepts capture challenges that, to some degree, are present in singular urban crises. However, the compounding of shocks increases their complexity, unpredictability, and persistence, thereby increasing concerns about ensuring effective and just urban governance. Below, we reflect on implications for research on the governance of compound urban crises.

The concept of unsettlement underlines interconnections between global dynamics and the politics of urban precarity. A CAS perspective explains how interventions within a city may generate useful synergies or unintended outcomes in another sector, location, or time (Coetzee et al. 2016), while the CUS scholarship underlines that any new shock is situated in a landscape of inequality and exclusion. There are two main directions of examination required to understand such interconnections. First, how do politics and relations in the city shape vulnerabilities to network interactions? Do particular ownership structures, management strategies, material configurations, or patterns of access to services and infrastructures affect exposure to global disruptions? If this is the case, how can governance arrangements be rearranged to reduce such vulnerabilities, particularly in ways that protect the most disadvantaged? Second, what are the links between transnational politics and urban risk? Policy and best practice for urban management circulate through international networks, lending legitimacy to certain programmes of action (e.g. renewable energy policy). Yet, such actions create risks and burdens for populations elsewhere (e.g. through material extraction and waste disposal), as witnessed, for example, through the phenomenon of sacrifice zones (Zografos and Robbins 2020). Calls have been raised to coordinate environmental actions across scales (Chan et al. 2015). Beyond the task of quantifying and monitoring impacts, we raise the question of how to build transnational to local solidarity to address invisibility and challenge legitimised notions of expendability (de Sousa Santos 2015) associated with communities at the receiving end of risks.

The challenge of unevenness draws attention to the reproduction of vulnerabilities in urban regions under pre-existing conditions of injustice. We identify two main directions of research required to unpack this challenge. First, there is a need to examine how existing urban governance systems promote unevenness, including through path dependencies built into decision-making processes and policy rationalities. In many metropolitan regions in the U.S., for instance, governance systems are designed to reinforce inclusion and exclusion and the fragmented governance system allows for regional inequity (Rosan 2016). Likewise, financial instruments capitalise on contingency, fluidity, and uncertainty in urban contexts and convert these conditions into value that is commodified and exchanged, thus shaping geographies of investment and exclusion across the city. Second, we need a greater understanding of the impacts of policy strategies designed to tackle compound crises, such as attempts to link pandemic recovery with environmental interventions through ‘green recovery’ packages. While the social justice implications of such initiatives are not yet known, it is clear that responses to urban disruption often exacerbate unevenness. For example, actions to reduce climate impacts in urban areas often reproduce capitalist logics and entrench crisis-prone modes of development (Long and Rice 2020). State-led action to reduce economic instability that is fixed in neoliberal policy serves to recreate rather than ameliorate economic shocks (Jones and Ward 2002). Infrastructure investments may also cement inequalities along the lines of racial oppression (Pulido 2017).

The concept of unbounding highlights the challenge of responding to crises of a constantly shifting and unpredictable character. We see two ways forward in research on this problem. The first relates to realising a commitment to knowledge pluralism, in recognition that there is no one way to understand compound urban crises. These phenomena cannot be reduced to matters of fact; they are better confronted as ‘matters of concern’ with contested boundaries between facts and values (Latour 2004). Rather than reaffirming a ‘monolithic’ idea of science, research should foster transdisciplinary knowledge co-production (Webb et al. 2018). This also requires rethinking who counts as a ‘stakeholder’, to ensure the inclusion of groups beyond the usual suspects. For instance, the technocratic design of climate policy reflects and privileges participation of dominant organisations, which reproduces social injustice (Malloy and Ashcraft 2020). In particular, everyday experiences need to be considered while examining the nature and implications of crises. For example, the Black Lives Matter movement has exposed how everyday violence is the norm for black communities, rather than the exception (Anderson 2017). Power relations define what counts as an urban crisis, often in terms of how crises threaten urban elites' privileges and demands for security. Second, as the boundaries of traditional sectors dissolve, further research is required to understand how policy strategies may tackle multiple interacting drivers of vulnerability. It is not clear, for example, what forms of intervention might address external causes of instability (e.g. global environmental change or international finance) and urban conditions that perpetuate inequality (e.g. capitalist modes of development, political exclusion, or racism). While this remains an open question, future research could seek to clarify the effectiveness of policy interventions that target equity, inclusion, and social wellbeing. This may be realised by addressing multiple conditions that cause vulnerability, for example, by creating access to healthcare, safe and affordable housing, financial security, legal status, or considering issues of recognition.

## Conclusions

We live in an age of compound urban crises; this is already significantly affecting the everyday lives of urban residents and has major consequences for urban governance. A key challenge is to make practical headway on compound urban crises without being paralysed by complexity. This encourages and requires reflexivity about unintended consequences of interventions. Yet, it also draws attention to co-beneficial actions and simultaneous interventions in multiple areas. Understanding how social-ecological problems are intertwined may be a step towards breaking down policy silos and adopting holistic political responses in a changing global environment.

While multiple crises co-exist, certain crises are elevated in news stories and many everyday crises remain invisible. Multiple points of view regarding what constitutes the most pressing form of disruption always co-exist and power relations determine which crises are presented as most urgent. We can only genuinely learn about experiences of crises through dialogue with those most affected by threat and uncertainty. At the same time, the need to draw on multiple views and experiences arises in an environment of political and social polarisation. As debates move towards extremes, there is little ground for collective deliberation and problem-solving. Likewise, rifts within academia limit conversations across disciplinary divide. This paper represents a bridging effort, employing CAS and CUS scholarship to develop three boundary concepts as an entry point for interdisciplinary discussion. We also identified parallels beyond these literatures, such as constructivist perspectives on policy studies. For example, the notion of punctuated evolution (Hay 2006) resonates with ideas of non-linear change in CAS, while the framing of equity in policy studies (Stone 1988) provides additional perspectives on unevenness. The core feature of boundary concepts is their ability to embrace diverse understandings without requiring consensus, allowing scholars to overcome the conceptual barriers that hamper knowledge production. We do not advocate Frankensteinian frameworks collating non-compatible forms of knowledge into unwieldy theoretical apparatuses. Rather, we argue that boundary concepts can cultivate an appreciation of how insights from other fields enrich and extend those of our own.

As we carefully map out the conceptual domain of compound crises, communities already respond to their impacts everyday, discovering what works through the application of lay expertise and learning by doing. Extended case studies are particularly useful in gaining insights from practice, serving as the empirical ‘holding ground’ for the theoretical anchor that boundary concepts represent. The storm we hope to navigate is the compelling problem of compound urban crises. Together, these ideas represent an initial communicative space to explore ways forward in a turbulent era.
